# Mr. Neyle, on Cow-Pox and Small-Pox Inoculation

**Published:** 1806-08

**Authors:** William Neyle

**Affiliations:** Piddletown, Dorset


### *To the Editors of the Medical and Phyfical Journal.*

GENTLEMEN,

AS the superiority of the vaccine or cow-pock over the small-pox, may perhaps be best seen by a comparative view view of their different effects; I think it may be of public utility to make known the result of the observations respecting both diseases, as they fell under my notice during a general inoculation, which took place here, and in the neighbouring parish of Burlston last month; in the first week of which I inoculated with variolous, or small-pox matter, 336 patients, of which number thirty had been vaccinated by myself last summer, and two, four years since; and four more by other gentlemen. These thirty-six were now variolated for their own satisfaction. I *now* also vaccinated twelve, of whom two were inoculated with small-pox matter within 48 hours after the insertion of the vaccine fluid; the other ten, with nine more, who had passed the cow-pock before, stood their chance, at present, without further inoculation.

The result has been as follows: of those variolated, viz. 300, although strictly dieted, well physicked, and, in general, highly and commendably attentive to all my directions, (which were strictly cooling and antiphlogistic), and although the weather has been favourable *for the season*, (a brisk North or North-East wind *generally* prevailing during the month) forty have had no more than a common sprinkling of pustules, giving a good deal of trouble to their friends; forty-five have had the disease so heavy, as to require constant attendance both by day and by night, during the eruptive fever and state of maturation, and have *all* been for a shorter or longer period blind; ten have been so dangerously ill, as to demand regular medical attendance, and have recovered with much difficulty, and in one or two instances, even against hope; and one has actually fallen a victim to the malignity of the disorder!

Whereas *all* (in number 57) who had been before, or were at this time vaccinated, escaped contagion from the small-pox, although they lived intermixed with those sick in that disorder, in the same village, under the same roofs, nay, in the same chambers with them: having themselves passed, what can hardly be termed a disease, without pain to themselves, or trouble to their friends; without attention to diet or regimen, and, what may be thought still better, without physic.

I am, &c.

*Piddletown, Dorset,*
*June 4, 1806.*

WILLIAM NEYLE.

